# Association between pharmacist-led telehealth services and improvements in cardiovascular outcomes among patients with cardiovascular risk factors: A scoping review

**DOI:** 10.1016/j.ijcrp.2023.200206

**Published:** 2023-08-22

**Authors:** Arinze Nkemdirim Okere, Aliyah Balogun, Angela Smith, Jade Stevens

**Affiliations:** College of Pharmacy and Pharmaceutical Sciences, Institute of Public Health, Florida A&M University, 1415 Martin Luther King Jr. BLVD, Tallahassee, FL, 32307, USA

**Keywords:** Telehealth, Pharmacist, Cardiovascular Risk Factors

## Abstract

**Background:**

Cardiovascular disease is the leading cause of death globally. Despite the effectiveness of lifestyle changes and recommended therapeutics, access to primary care and treatments to improve cardiovascular risk-factors (CRFs) remains challenging. Pharmacists and telehealth services have been proposed as potential solutions to overcome these barriers.

**Methods:**

PubMed, OVID, and CINAHL databases were searched from January 2006 to March 2023. The primary outcomes were changes from baseline in systolic/diastolic blood pressure, glycated hemoglobin (A1c), cholesterol levels, and adherence to any patient counseling. Only studies conducted in the United States and Canada were included in the review.

**Results:**

Of 110 screened bibliographic records, 14 studies were included in the review. The pharmacist-led telehealth interventions included medication therapy management, medication reviews, and counseling on lifestyle changes. Nine studies reported significant improvements with intervention, 7 studies on CRFs and 2 studies on medication adherence at the 12-month follow-up, when pharmacist-led telehealth services were compared to usual care or historical data (p < 0.05).

**Conclusion:**

This scoping review provides evidence for continued support to the development and implementation of pharmacist-led telehealth services in primary cardiovascular care. The findings suggest that pharmacist-led telehealth interventions can improve cardiovascular outcomes and adherence to drug and non-drug therapy among patients with CRFs. However, because of lack of published randomized clinical studies on patients with CRFs residing in underserved communities, future directions in research should focus on exploring the implementation of pharmacist-led telehealth services in rural or underserved communities, utilizing various payment models to enhance accessibility and feasibility.

## Highlights

• Pharmacist-led telehealth services for cardiovascular care yield comparable outcomes to in-person consultations in improving primary and secondary cardiovascular outcomes.
• TPharmacist telehealth is associated with improved cardiovascular risk factors like diabetes, hypertension, and dyslipidemia.
• Patients with cardiovascular disease living in rural areas can benefit from pharmacist telehealth by reducing the need for in-person visits.
• Robust studies examining pharmacist-led telehealth’s impact on rural cardiovascular patients are lacking..
• Future research should explore the use of diverse payment models to make telehealth accessible to underserved or rural areas.

## 1 Introduction

Despite ongoing efforts to reduce the risk of cardiovascular disease, heart disease remains the leading cause of death in the United States and worldwide [1]. Heart disease accounted for 697,000 deaths in the United States (US) in 2020, constituting one-fifth of all deaths.^2,3.^ Also, its socio-economic burden is high. The economic cost of heart disease in the United States reached an estimated sum of $229 billion during the years 2017–2018 [2,3]. Thus, these reports underscore the persistent need for effective interventions and policies aimed at improving access to primary cardiovascular care for combating this pervasive and potentially fatal health condition.

Despite the recognition that increasing physical activity, making lifestyle changes, and adhering to recommended therapeutics can reduce cardiovascular risk factors associated [[4], [5], [6]], access to fundamental primary cardiovascular care and treatments to improve cardiovascular risk factors continues to be a significant barrier. Therefore, to enhance access to primary cardiovascular care, we proposed that an optimal approach would entail integrating a clinical pharmacist as a frontline healthcare professional in primary care and utilizing telehealth services. These approaches have been studied separately and their combination can be a unique model to adapt.

In the first approach to improve cardiovascular health outcomes through the utilization of community pharmacist in the primary care team, a systematic review by Martínez-Mardones et al. (2019) revealed that pharmacist-led medication reviews were effective in improving cardiovascular health risk factors [7]. However, one limiting factor for the adoption of this approach in primary care was the associated costs of supporting an outpatient clinical pharmacist.

In the second approach that involves the use of telehealth services in clinical settings to enhance access to care, utilization of telehealth services was reported to have similar positive effects as in-person clinical consultations, with the added benefit of increasing access to necessary primary care. A meta-analysis conducted by Kuan et al. (2022) revealed that telemedicine has similar efficacy as clinic-based consultation in reducing the risk of cardiovascular-related mortality and hospitalization in patients with heart failure [8]. Therefore, telemedicine is a potential tool for improving access to care while maintaining optimal health outcomes. However, the potential limitation to its adoption in the US is limited availability of reimbursement issues and resistance to change [9,10].

Based on the evidence for the aforementioned two approaches, we then hypothesized that integration of pharmacist and telehealth care services can provide protective benefits among patients with cardiovascular risk factors, at least similar to in-person clinical consultation, with an underscoring positive resultant effect of increasing access to cardiovascular care. This hypothesis is supported by two studies conducted by Margolis et al. [11] and Niznik et al. [12] These two studies highlight the crucial role of pharmacist-led telehealth services in outpatient settings to optimize patient health outcomes. There is a need to strengthen the evidence on the effectiveness of pharmacist-led telehealth services in improving access to primary cardiovascular care in the United States. To achieve this goal, we will conduct a thorough review of articles primarily carried out in North America, focusing specifically on the United States and Canada. These countries share sociocultural characteristics and have comparable pharmacist education and training.

The aim of this paper is to review the association between pharmacist-led telehealth services, cardiovascular risk factors, and adherence to drug and non-drug recommendations. This review is critical because preventing cardiovascular risk factors and improving adherence are pivotal steps in avoiding major cardiovascular death and morbidity [4,13]. To the best of our knowledge, such a review has not yet been conducted. Thus, the primary objective of our scoping review is to provide evidence to strengthen public health care policies to support the development and implementation of pharmacist-led telehealth services to enhance access to primary cardiovascular care.

## 2 Methods

This scoping review adheres to the PRISMA-SCR (PRISMA extension for Scoping Reviews) guidelines to provide a critical overview of the association between cardiovascular health outcomes with pharmacist-led telehealth. The scoping review framework developed by Arksey and O'Malley, with recent advancements by Levac et al., was adapted for this review [14,15].

### 2.1 Identifying research questions

The main research question is: Is there evidence to support the association between pharmacist-led telehealth services and improvements in cardiovascular health outcomes among patients with cardiovascular risk factors?

### 2.2 Identifying relevant studies

A comprehensive literature search was conducted on articles published from January 2006 to March 2023, indexed in the US National Library of Medicine's PubMed, OVID, and CINAHL databases. The following keywords were used for PubMed search: (“pharmacist and telehealth and cardiovascular disease”), (“pharmacist and telemedicine and cardiovascular disease”), [(“tele*" or “online”) and (“cardiovascular or hypertension or diabetes or dyslipidemia” and Pharmacist)], and [(“tele” or “online” or “monitoring”) and (cardiovascular or hypertension or diabetes or dyslipidemia) and (“Pharmacist-lead” or “Pharmacist-led")], restricted to clinical trials and randomized clinical trials. For OVID, and CINAHL search we used the following: (“pharmacist and telehealth or telemedicine and cardiovascular disease”), and [(“tele” or “online” or “monitoring”) and (cardiovascular or hypertension or diabetes or dyslipidemia) and (“Pharmacist-lead” or “Pharmacist-led")]. Clinical trials registered in clinicaltrials.gov were also reviewed for randomized controlled studies using the keyword “pharmacist telehealth cardiovascular disease.” These broad keywords were selected to ensure the identification of all relevant studies.

### 2.3 Study selection process

Bibliographic records obtained from the literature search were initially screened for duplicates and removed. Four reviewers (AN), (AB), (AS) and (JS) assessed the eligibility of the resulting articles based on title/abstract and full-text screenings. Only articles published in the English language were included for clinical review. There was no disagreement during this process.

### 2.4 Inclusion and exclusion

The studies included in this scoping review were selected based on the following inclusion and exclusion criteria.

#### 2.4.1 Study design

Randomized controlled and non-randomized controlled trials. Descriptive or case reports and editorials were excluded.

#### 2.4.2 Population

The review included studies that involved patients with cardiovascular risk factors in the US or Canada. Studies conducted outside the US or Canada were excluded.

#### 2.4.3 Interventions

Studies evaluating pharmacist-led telehealth interventions on patients with cardiovascular risk factors were included. Telehealth interventions provided only by physicians or nurses, or other healthcare providers were excluded.

#### 2.4.4 Comparators

Studies that compared the intervention group (pharmacist-led telehealth services) with either standard care or historical data were included.

#### 2.4.5 Outcome

The primary outcome measured changes from baseline in systolic/diastolic blood pressure, glycated hemoglobin (HbA1c), and cholesterol levels (LDL-C, HDL-C, triglycerides, and total cholesterol), as well as adherence to any patient counseling, whether drug or non-drug related.

### 2.5 Data extraction and synthesis

The data from the selected papers were extracted and synthesized using a predefined extraction form (refer to Table 1). Consistent with the scoping review framework developed by Arksey and O'Malley, the quality and strength of evidence of each study were not evaluated.

**Table 1:** Selected studies on pharmacist-led telehealth in patients with cardiovascular risk factors.

Citation (Country)	Study Design	Communication Type	Population Description	Number Of Participants	Duration	Intervention	Control Group	Primary Outcome	Results
Margolis et al. [11] **(USA)**	Clustered randomized	Telephone	Adult patients with moderately severe hypertension (BP ≥ 150/95 mm Hg):	3071	12 months	Pharmacist led telehealth visit	Clinic based in-person visit	Change in Systolic Blood pressure	• Systolic Blood pressure decrease in both groups ((difference: 0.8 mm Hg [95% CI, −2.84 to 1.32]) with no statistical difference (P > 0.05)
Bosworth et al. [16] **(USA)**	Randomized Control Trial	Telephone	Veterans with poorly controlled hypertension and/or hypercholesterolemia	428	12 Months	Clinical Pharmacist specialist Intervention with behavioral and medication management for 12 months	Educational Material alone	Improvement in CVD risk score at 6 and 12 months	• At 12 months, there both groups experienced similar decrease in systolic Blood pressure (SBP), total cholesterol (TC), LDL-cholesterol, and HbA1c (P > 0.05) • Intervention group experienced higher HDL-cholesterol than control group (Mean difference [MD] = 7.3, 95% CI 0.4–14.9
Milani et al. [17] **(USA)**	Randomized Control Trial	Telephone, Smart phone device	Patients diagnosed with hypertension at the Ochsner Health System who had elevated blood pressure at 3 of their most recent physician visits within the previous 18 months	556	3 months	Pharmacist provided medication, lifestyle and disease state counseling and created care plans with participants	Usual Care	Proportion of participants with controlled blood pressure at 90 days	• More participants in the intervention group achieved blood pressure goal (71%) versus participants in the usual care group (31%): (P < 0.001)
Salvo et al. [18] **(USA)**	Retrospective Cohort Study	Telephone	Patients with type 1 or type 2 diabetes using insulin therapy and receiving primary care services at the St. Louis County Department of Health	126	18 months	Pharmacist Managed Diabetes Care	Standard care (Diabetes management by primary care physician	Change in A1C between the 2 groups from index date to 3, 6, 9, 12, and 18 months, and at study end	• At 3 months change in A1C versus the standard care group (−0.64% vs −0.11%, p = 0.022) • At 6 months, change in A1C versus the standard care group (−1.0% vs −0.21%, p = 0.009) • At 9 months, change in A1C versus the standard care group (−1.2% vs −0.30%, p = 0.004), • At 12 months, change in A1C versus the standard care group (−1.2% vs −0.24%, p = 0.004) • At 18 months, change in A1C versus the standard care group (−1.27% vs −0.17%, p = 0.001) • At study end, change in A1C versus the standard care group (−1.3% vs −0.18%, p = 0.001)
Margolis et al. [19] **(USA)**	Clustered randomized	Telephone	Adult patients with moderately severe hypertension (BP ≥ 140/90 mm Hg)	450	12 months	Pharmacist led telehealth visit	Clinic based in-person visit	Proportion of patients who achieved blood pressure control	• At patient visit at 6 and 12 Months, proportion of patients were higher in intervention group 57.2% (95% CI, 44.8%–68.7%) than usual care group 30.0% (95% CI, 23.2%–37.8%) [p < 0.001] • At patient visit at 6, 12 and 18 Months, proportion of patients were higher in intervention group 50.9% (95% CI, 36.9%–64.8%) than usual care group 30.0% 21.3% (95% CI, 14.4%–30.4%) [p < 0.002]
Magid et al. [20] **(USA)**	Pragmatic, Randomized controlled trial	Heart360-supported web	Patients between 18 and 79 years of age with a diagnosis of hypertension and have the following: • 2 most recent clinic BP readings were above goal (systolic BP [SBP] ≥140 mm Hg or diastolic BP [DBP] ≥90 mm Hg or, for those with diabetes or chronic kidney disease (CKD), SBP ≥130 mm Hg or DBP ≥80 mm Hg); • At least 3 antihypertensive medications • Have a primary care provider who worked at 1 of the 10 participating clinics	326	6 months	Heart360 web, Telephone or email	Usual care	Proportion of patients who achieve A1C goal at 6 months.	• Higher proportion of patients in intervention group (58%) achieved goal than the usual care group (42%; *P* < 0.001)
Peasah et al. [21]	Randomized	Telephone	Patients with diabetes with A1C at least 7.0%	78	12 weeks	Pharmacy students-initiated telephone	Usual care (no pharmacist or pharmacy students intervention)	Change in A1C from baseline to week 12	• Mean paired difference showed no difference between the 2 groups • Multiple regression showed higher end of study A1C level in the control group than intervention group (0.5547, p = 0.002)
Green et al. [22] **(USA)**	Randomized Control Trial	Telephone visit, Web training and communication	Patients 25–75 years with uncontrolled essential hypertension and Internet access	778	12 Months	• Home blood pressure monitoring and secure web training only. • Home blood pressure monitoring, web training and pharmacist care management using web communication	Usual Care	• Change in systolic and diastolic blood pressure at 12 weeks • Percentage of patients with controlled blood pressure at 12 months	• Significant number of patients who achieved blood pressure control were higher in patients who received web training plus pharmacist telephonic visits and home blood pressure monitoring (RR 1.84 [95% CI, −1.48 – 2.29]; p < 0.001) when compared to usual care.
Lauffenburger et al. [23] **(USA)**	Randomized clinical trial	Telephone	Uncontrolled type 2 diabetes	1400	12 month Follow-up	Pharmacist telephone visit	Usual care	• Change in A1C from baseline • Change in medication adherence	• No significant difference in mean change in A1c from baseline to follow-up (between group difference: +0.04, 95% Confidence Interval [CI]: 0.22, 0.30)). • Propensity-score matched ‘as-treated’ analyses the mean pre- to post-change in A1c was [(-0.48 (1.73) for control and −0.96 (SD: 1.69) for intervention (difference: 0.48, 95%CI: 0.91, −0.05)] • No change in medication adherence
Ma et al. [24] **(USA)**	Randomized Control Trial	Telephone	Patients aged 30–85 years who have coronary heart disease.	689	9 Months	• Multiple telephone counseling sessions provided by a pharmacist + computer-based tracking for follow up	Usual Care	Percentage of patients with an LDL-C level <100 mg/d	• No difference in cardiovascular medication adherence and proportion of patients who achieved LDL-C goals (P = 0.29).
Cohen et al. [25] **(USA)**	Open label Randomized Control Study	Telephone	Veterans with type 1 or type 2 diabetes, an A1C ≥ 7.5%, diagnosis of depression, and access to a landline phone were invited to participate	30	6 Months	Pharmacist-led telehealth	Nurse-led telehealth	• Change in diabetes and depression medication adherence in the intervention group versus the control group at 6 months • Mean change in A1C from baseline	• There was statistical significance improvement of cardiovascular medication adherence with pharmacist-led telehealth (14.0; 95% CI 0.4 to 27.6) compared to nurse-led telehealth (10.3; 95% CI –10.0 to 30.6); P < 0.05) • No change statistical difference in mean change in A1C from baseline between pharmacist and nurse-led telehealth groups
Mohan et al. [26] **(USA)**	Randomized controlled trial	Telephone based motivational interview	Patients with hypertension enrolled in Texas Medicare Advantage Plan	720	12 months	Telephone outreach and counseling by a pharmacy student (Under the supervision of preceptors)	Usual care	Adherence to ACEI or ARB	• Within 6 months: No significant difference between Intervention patients versus control patients (OR 1.31 (0.94–1.83) • Within 12 months: Intervention patients had better adherence to ACEIs/ARBs than controls (OR: 1.46; 95% CI 1.05–2.04)
Odegard et al. [27] **(USA)**	Randomized Control Trial	Telephone	Patients with diabetes taking oral antidiabetic medications	265	12 months	Telephone-initiated adherence support (using “4 As”	Usual care	Medication possession ratio at 6 and 12 months	• At 6 months: MPR (for intervention group) improved from 0.90 to 0.92 (P = 0.16) • At 12 months: MPR (for intervention group) improved from 0.85 to 0.90 (P < 0.01) • No Change in MPR in control at 6- and 12-month period
Alsabbagh et al. [28] **(Canada)**	Randomized Control Trial	Telephone	Patients 30 years and older who were hospitalized due to ACS, revascularization, or revascularization surgery within the past 3 months and who had been prescribed a new cardiovascular medication	95	6 months	Telephone outreach and counseling by a pharmacist	Usual Care	Mean change in cardiovascular medication adherence (defined using Medication Possession ratio [MPR])	• No difference between the usual care and intervention group. The MPR of at least 80% to new cardiovascular medication was 82.6% and 79.2% in pharmacist-intervention and usual care groups, respectively, (P = 0.44)

## 3 Results

**Fig. 1:**
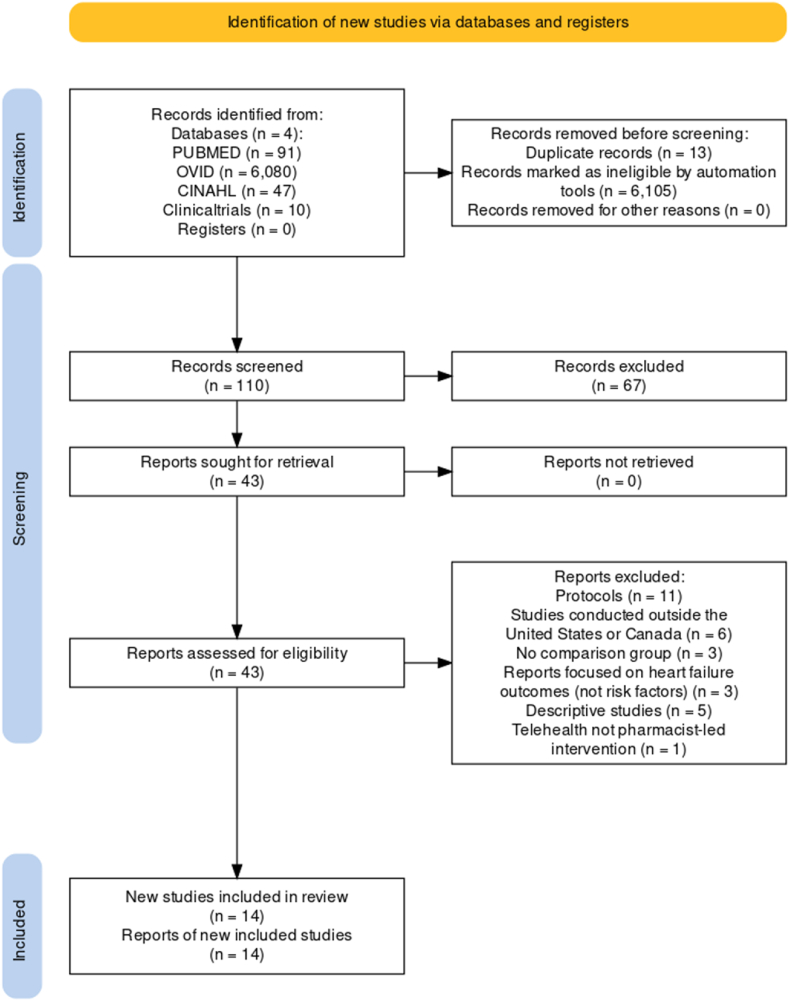
Reference: Haddaway NR, Page MJ, Pritchard CC, McGuinness LA. *PRISMA2020*: An R package and Shiny app for producing PRISMA 2020-compliant flow diagrams, with interactivity for optimised digital transparency and Open Synthesis. Campbell Syst Rev. 2022 Mar 27; 18 (2):e1230. https://doi.org/10.1002/cl2.1230. PMID: 36911350; PMCID: PMC8958186.

We initially searched the databases and found a total of 6228 articles, which were reviewed for relevance and duplication. After screening out duplicates, we reviewed 110 studies for inclusion/exclusion criteria. Sixty-seven studies were eliminated based on their titles and abstracts. We then screened the remaining articles for pharmacist involvement (inclusion) and study protocols and rationales (exclusion), leading to the exclusion of 28 articles. During data extraction, we excluded two more article, leaving us with a total of 14 eligible articles for review (see Fig. 1 for PRISMA flowchart). Only studies conducted in the United States (n = 13) and Canada (n = 1) were selected for review. Fourteen out of eleven studies were prospective randomized controlled trials and only 1 was a retrospective analysis. We categorized the studies that met our inclusion criteria into two categories: 1) Cardiovascular risk factors and 2) Adherence to cardiovascular medications/patient counseling.

### 3.1 Cardiovascular risk factors

In the evaluation of pharmacist-driven telehealth interventions on cardiovascular risk factors, nine studies focused on blood pressure, HbA1c and cholesterol [11,[16], [17], [18], [19], [20], [21], [22], [23]]. Only one of those studies evaluated effect on body mass index (BMI) [16].

#### 3.1.1 Selected study reports

Bosworth et al. [16]observed the difference in CVD outcomes between the pharmacist delivered telephonic based intervention versus education control only. By 12 months, there was similar decline in CVD risk between the CPS intervention (−3.3% in intervention; 95% Confidence Interval [CI] −4.9 to −1.8) and Control group (−3.0%; 95% CI -4.5 to −1.5). Decline in BMI, systolic blood pressure and HbA1c were observed in both study groups, but differences observed were not statistically significant. However, they did observe that the intervention group did have significant increase in HDL-cholesterol when compared to the control group.

Milani et al. [17] study aimed to determine the proportion of patients who achieved controlled blood pressure within 90 days. Blood pressure levels improved significantly in both the digital-medicine and usual-care groups, but at 90 days, 71% of patients in the digital-medicine group achieved blood pressure control compared to 31% of the usual-care group (P < 0.001). The digital-medicine group had a significantly higher number of blood pressure recordings in their electronic medical record.

Salvo et al. [18] evaluated the change in HbA1c levels between pharmacist management delivered telephonically and standard of care groups at different time points. The pharmacist management group showed a significant difference in mean change in HbA1c levels at all time points compared to the standard-of-care group (P < 0.004). The pharmacist group had a higher number of patients at HbA1c goal at study end (20.3% vs 14%, p = 0.48), despite having fewer patients at HbA1c goal at baseline.

Margolis et al. [19] conducted a study comparing pharmacist-led telehealth clinics to in-person clinic visits for blood pressure control. The primary outcome was the proportion of patients with controlled blood pressure at both 6- and 12-month clinic visits. Among the 380 patients attending both visits, 57.2% in the telemonitoring intervention group and 30.0% in the usual care group had controlled BP (P = 0.001). For the 362 patients attending all clinic visits at 6, 12, and 18 months, 50.9% in the telemonitoring intervention group and 21.3% in the usual care group had controlled BP at all visits (P = 0.002). In 2022, Margolis et al. [11] reported their study comparing the effect of clinic-based, in-person visits with pharmacist-led telehealth care on adult patients with moderately severe hypertension (BP ≥ 150/95 mm Hg). Over a 12-month follow-up period, both groups experienced a significant reduction in BP to <140/83 without significant difference in systolic BP change between the two groups (P < 0.05).

Finally, Magdid et al. [20] study aimed to compare the effectiveness of a pharmacist led HBPM intervention (Heart360 website supported intervention) with usual care for patients with uncontrolled hypertension. The primary outcome was the proportion of patients achieving their BP goal at the 6-month clinic visit. After 6 months, the HBPM group had significantly lower mean BPs and a significantly higher proportion of patients achieving their BP goal compared to the UC group. In the subset of patients with DM and CKD, the proportion of patients achieving BP goal was also higher in the HBPM group. The adjusted risk ratio for the HBPM group was 1.5 (95% CI, 1.2–1.9) for all patients and 2.5 (95% CI, 1.6–3.8) for the subset with DM and CKD.

Peasah et al. [21] conducted a prospective, randomized, pilot study to assess the effectiveness of a telephone follow-up intervention for diabetic patients with HbA1c levels of 7% or higher. Trained pharmacy students, under pharmacist supervision, conducted the telephonic intervention. The primary focus was to examine the change in HbA1c levels at 12 weeks from baseline. The results showed that there was no significant differences in the mean HbA1c at 12 weeks between the two groups. However, the control group exhibited higher end of study HbA1c values than the intervention group (0.5547, P = 0.002), after adjusting for baseline HbA1c levels and other demographic variables.

Green et al. [22] observed that a combination of home blood pressure monitoring and web-based training alone did not improve blood pressure control significantly but did result in a modest reduction in systolic blood pressure. However, adding web-based pharmacist care to the combination of home monitoring and web training resulted in significantly more patients with controlled blood pressure (56% [95% CI 49%–62%]) compared to those receiving usual care (31% [95% CI 25%–37%]) or only home monitoring and web training (36% [95% CI 30%–42%] p < 0.001). Finally, Lauffenburger et al. [23] examined the impact of telephone-based intervention on patients with poorly controlled diabetes. In the study, they found a small difference in the change in HbA1c levels between control and intervention subjects, with a mean change of −0.79 ((standard deviation [SD]: 2.01) and −0.75 (SD: 1.96), respectively. The observed difference was not statistically significant. However, in a propensity-score matched analysis comparing similar patients in each group they observed a significant between-group difference of −0.48 (95% CI: 0.91, −0.05) favoring the intervention group [mean change in HbA1c: for control, −0.48 SD: 1.73 versus for intervention, −0.96 SD: 1.69)].

### 3.2 Adherence to cardiovascular medications/counseling

In this section, only 5 unique studies evaluated the effect of pharmacist driven telehealth on adherence on medication use and preventive patient-care counseling [[24], [25], [26], [27], [28]]. Three studies that reported effect on cardiovascular risk factors also evaluated effect on adherence [18,20,23]. Selected Study Reports were categorized to medication adherence and preventive care.

#### 3.2.1 Medication adherence

Ma et al. [24] study examined the effect of a pharmacist delivered intervention (PI) on cholesterol levels and medication adherence in comparison to usual care (UC). The primary outcome was the percentage of patients with LDL-C levels under 100 mg/dl, and the secondary outcome was the proportion of statin medication taken as measured by CMA. At 12 months, there was no significant difference between the PI and UC conditions in achieving the goal LDL-C level or medication adherence. The levels of total cholesterol, HDL-C, LDL-C, and triglycerides were similar in both conditions. Additionally, there were no statistical differences in the use of beta-blockers and ACE inhibitors between the two groups. Similarly, Cohen et al. [25] examined the effect of pharmacist led telehealth when compared to nurse-led telehealth. They observed that at the six-month follow-up, the pharmacist-led telehealth group showed significant improvements in cardiovascular medication adherence (14.0; 95 CI [0.4–27.6], compared to baseline. Also, Mohan et al. [26] conducted a study to assess the impact of a phone-based motivational interview (MI) intervention on improving adherence and persistence to ACEIs/ARBs among nonadherent patients with hypertension and diabetes enrolled in Medicare. The intervention was carried out by trained fourth-year pharmacy students under the supervision of pharmacists. The main focus was to determine adherence to ACEI/ARB medication at 6 and 12 months after the implementation of the MI intervention. In each case, the results showed that patients in the intervention group had a significantly higher adherence rate compared to the control group (P < 0.05). Similarly, Odegard et al. [27] assessed the impact of utilizing the “4 A's" (ask, advise, assist, and arrange) model via telephonic intervention. The study revealed that the intervention group experienced a statistically significant improvement in medication adherence during the 12-month follow-up as evidenced by a higher mean medication possession ratio [MPR] exceeding 80% in the intervention group compared to the control group (odds ratio 4.77, 95% CI 2.00–11.40). However, no statistically significant difference in MPR was observed between the intervention and control groups during the 6-month follow-up period.

In contrast to the observations noted in the 12-month follow-up period, but mirroring the findings observed during the 6-month follow-up, Alsabbagh et al. [28] did not see any difference in change in mean cardiovascular medication adherence MPR between the intervention and usual care groups. However, the observed result was limited to the small sample size (n = 15 patients per group). Notably, Magdid et al. study [20], with a larger population (n = 164 per group), did show no significant difference in the mean MPR adherence score over the 6-month study period between the groups, with a score of 0.86 in the intervention group and 0.87 in the control group (P = 0.93). Similarly, Lauffenburger et al. [23] did not see statistically significant differences in medication adherence.

#### 3.2.2 Preventive care

Salvo et al. [18] evaluated the likelihood of adhering to ADA recommended preventive care measures. The study found that patients in the intervention group were more likely to complete ADA recommended preventive care measures compared to the standard-of-care group. The intervention group had a statistically significant difference in completing annual foot and eye examinations, receiving influenza and pneumococcal vaccines, and having a microalbumin screen, with more than half of the patients in the intervention group completing these measures (P < 0.05).

## 4 Discussion

Our scope review aimed to examine existing literatures on the association between pharmacist-led telehealth services and improvements in cardiovascular outcomes among patients with cardiovascular risk factor. Approximately 78% (7 out of 9) of the reviewed reports on the effect of pharmacist-led telehealth services on cardiovascular risk factors demonstrated an association with improved outcomes. Of note, we found that telehealth services provided by pharmacists for cardiovascular care are mostly similar to in-person clinic consultations in improving primary and secondary cardiovascular outcomes. This results in a decrease in the number of in-person clinic visits, which is particularly appealing to patients living in rural communities or difficult to reach areas.

Regarding adherence to drug and non-drug therapy counseling, out of the 8 studies analyzed, three did not reveal statistically significant improvements in medication adherence. During the 12-month follow-up, positive results were observed, while no significant effect was seen in the 6-month follow-up period [26,27]. Therefore, the absence of statistically significant differences observed in the three studies may be attributed to two factors: either a failure to effectively address and minimize the various barriers linked to medication adherence, or insufficient time for the intervention to significantly impact the psychological barrier associated with medication adherence. We recognize that certain social determinants of health that impact medication adherence may not be effectively addressed through telehealth alone such environment, employment etc. Our hypothesis posits that by taking into account the influence of these determinants and implementing telehealth services in an optimal manner, we can potentially mitigate the negative effects on medication adherence. Further research is needed to gain a comprehensive understanding of how telehealth services can be utilized to minimize the adverse impact of social determinants of health, extending beyond access to care. Notably, achieving this objective will require a healthcare approach that spans multiple sectors, effectively integrating services to address diverse social determinants of health [29].

Providing health equity in telehealth means making changes in digital literacy, technology, and analytics, and can help telehealth providers reach underserved communities where cardiovascular disease is the leading cause of death [30]. Our findings have important public health implications, especially as the COVID-19 pandemic has highlighted the need for remote care options. While the US Department of Health and Human Services has taken steps to expedite the adoption and awareness of telehealth [31], there is a need to extend the use of telehealth to cardiovascular diseases and include pharmacists to improve care among high-risk patient populations.

### 4.1 Gap in literature and future directions

Based on our extensive literature review and current knowledge, no randomized clinical trials have been reported thus far that evaluate the impact of pharmacist-led telehealth services in primary care specifically targeting cardiovascular risk factors. This is a significant gap considering that heart disease remains the primary cause of mortality among individuals residing in rural or underserved communities. It is worth noting that Clark et al. [31] has shown the feasibility and positive impact of telehealth services on patients with hypertension residing in rural or underserved communities. Consequently, the inclusion of outpatient clinical pharmacists within this care model should be regarded as a promising avenue for future exploration, particularly for extending support to patients in rural or underserved areas.

### 4.2 Strengths

One strength of our study is that most of the studies selected for review were randomized controlled trials, which provide the highest level of evidence in clinical research.

### 4.3 Limitations

The inclusion of studies solely published in the English language and in the literature may have introduced a risk of publication bias. Additionally, there has not been a consistent telehealth approach in improving primary cardiovascular care, which may have led to differences in outcomes we reported on. Despite this limitation, there is a consistent message pointing towards the clinical benefits of pharmacist-led telehealth services among patients with cardiovascular risk factors.

Telehealth presents a promising solution to the shortage of providers and clinical pharmacists available to treat patients with cardiovascular risk factors in rural communities. Despite the potential benefits, it is important to note that many health plans in the United States do not cover pharmacist-led telehealth services. Therefore, demonstrating how the aims of the telehealth program align with the clinic's strategic vision is crucial for obtaining leadership support [28]. Fortunately, as evidenced from results emanating from studies unrelated to cardiovascular management [32,33], health plans may be attracted to the potential cost savings associated with more virtual visits - which eliminates the need for physical clinic offices or community pharmacies. Additionally, patients may be drawn to the idea of receiving optimal primary cardiovascular care from the comfort of their homes, saving time and travel costs typically associated with seeking in-person care. Consequently, improving patient satisfaction with the care they receive [34,35].

According to a report by the Centers for Disease Prevention and Control [36], the implementation of telehealth programs to address cardiovascular risk factors in low-income communities may face barriers, such as unreliable internet access and resistance from older patients who lack the necessary skills to use telehealth technologies. In addition, differences in telehealth adoption were observed among patients of different races and ethnicities. However, healthcare professionals perceived that telehealth could help reduce no-show rates, increase medication adherence, and improve patient reach. Telehealth was also perceived as an effective solution for patients who faced transportation and time barriers to accessing in-person healthcare services [29,36]. To overcome these barriers and enhance facilitators, public health policy must change to promote monetary incentives for expanding pharmacist-led telehealth services for primary cardiovascular care, and to improve the expansion of health and digital literacy and promotion.

### 4.4 Future perspective

The rapid advancements in remote telemonitoring, digital medicine, and mobile health technologies are expected to drive the integration of these cutting-edge tools into pharmacist-led telehealth services. This integration holds great potential for enhancing access to cardiovascular care.

Finally, based on our critical examination that considered differences in implementation strategies, bias, and sample sizes, the effect of pharmacist-led telehealth services yielded mixed results. Nevertheless, it is noteworthy that the intervention did not result in worse outcomes when compared to usual care. In conclusion, the model of pharmacist-led telehealth services has the potential to enhance patients’ access to primary cardiovascular risk factors while optimizing health outcomes, particularly among rural and underserved populations.

## Funding

None.
